# *Impatiens bokorensis* (Balsaminaceae), a new species from Cambodia

**DOI:** 10.3897/phytokeys.77.11345

**Published:** 2017-02-07

**Authors:** Seong-Hyun Cho, Bo-Yun Kim, Han-Sol Park, Chhang Phourin, Young-Dong Kim

**Affiliations:** 1 International Biological Material Research Center, Korea Research Institute of Bioscience and Biotechnology, 125 Gwahak-ro, Yuseong-gu, Daejeon 34141, Republic of Korea; 2 Department of Life Science, Hallym University, 1 Hallymdaehak-gil, Chuncheon-si, Gangwon 24252, Republic of Korea; 3 Forestry Administration, 40 Preah Norodom Blvd, Phnom Penh, Kingdom of Cambodia

**Keywords:** Phnum Bokor National Park, Endemic species, *Impatiens*, Cambodia

## Abstract

*Impatiens bokorensis*, a new species of family Balsaminaceae from Phnum Bokor National Park in southwestern Cambodia, is described and illustrated. The species is similar to *Impatiens patula*, but is readily distinguished by the orbicular-obovate dorsal petal, shorter pedicels and larger seeds.

## Introduction

Balsaminaceae is a family consisting of about 1,000 species mainly distributed in tropical Africa, Madagascar, southern India and Sri Lanka, the eastern Himalayas and southeastern Asia and is absent from Australia and South America (Song et al. 2003, Yuan et al. 2004, APG III 2009). This family includes annual or perennial herbs (more or less succulent) to sub-shrubs. It is distinguished from other families by strongly zygomorphic flowers with a spur on the adaxial sepal and a fleshy explosive-dehiscent capsule (Chen et al. 2007, APG III 2009). It comprises two genera: *Hydrocera* Blume ex Wight & Arn. (monotypic) and *Impatiens* L. with the most species.

*Impatiens* is classified into two subgenera (subgenus Impatiens Warb. and subgenus Acaulimpatiens Warb.) based on the presence or absence of cauline leaves. These two subgenera comprise 14 sections (subgenus Acaulimpatiens, two sections and subgenus Impatiens, 12 sections) mainly segregated by phyllotaxy, inflorescence and spur characters (Warburg and Reiche 1895, Utami 2009). Nonetheless, in a recent molecular phylogenetic study based on a nuclear ribosomal internal transcribed spacer (ITS) and plastid atpB-rbcL and trnL-F (Yu et al. 2015), *Impatiens* was classified into two subgenera (subgenus Clavicarpa S.X. Yu ex S.X. Yu & Wei Wang and *Impatiens*) with the subgenus Impatiens composed of seven sections (sect. Semeiocardium, sect. Racemosae, sect. Fasciculatae, sect. Tuberosae, sect. Scorpioidae, sect. Uniflorae and sect. Impatiens).

In Indochina, there are around 120 species of *Impatiens* and the present count includes approximately 40 species from Vietnam (Tardieu 1944, Ho 1999, Vietnam Plant Data Center 2016), around 60 species from Thailand (Grey-Wilson 1971, Shimizu 2000, Shimizu and Suksathan 2004, Chayamarit et al. 2006, Suksathan and Triboun 2009, Ruchisansakun et al. 2014) and 18 from Laos (Tardieu 1944, Newman et al. 2007, Newman 2008, Souvannakhoummane and Suksathan 2015). In Cambodia, at the beginning of the 20th century, six species were described, namely *Impatiens cardiophylla* Hook.f., *Impatiens diffusa* Hook.f., *Impatiens notoptera* Hook.f., *Impatiens relaxata* Hook.f., *Impatiens vagans* Hook.f. and *Impatiens zygosepala* Hook.f. (Hooker 1908, 1909a, 1909b, 1911), whereas eight species are reported in the present account (Cho et al. 2016).

Except for *Impatiens balsamina* and *Impatiens cardiophylla*, most species in Cambodia have been considered endemic species, with *Impatiens relaxata*, *Impatiens vagans* and *Impatiens zygosepala* restricted to a local area with only a very small number of specimens. There is a need to re-evaluate and resurvey areas of the previous collection of specimens through a detailed taxonomic study of each species.

**Figure 1.**
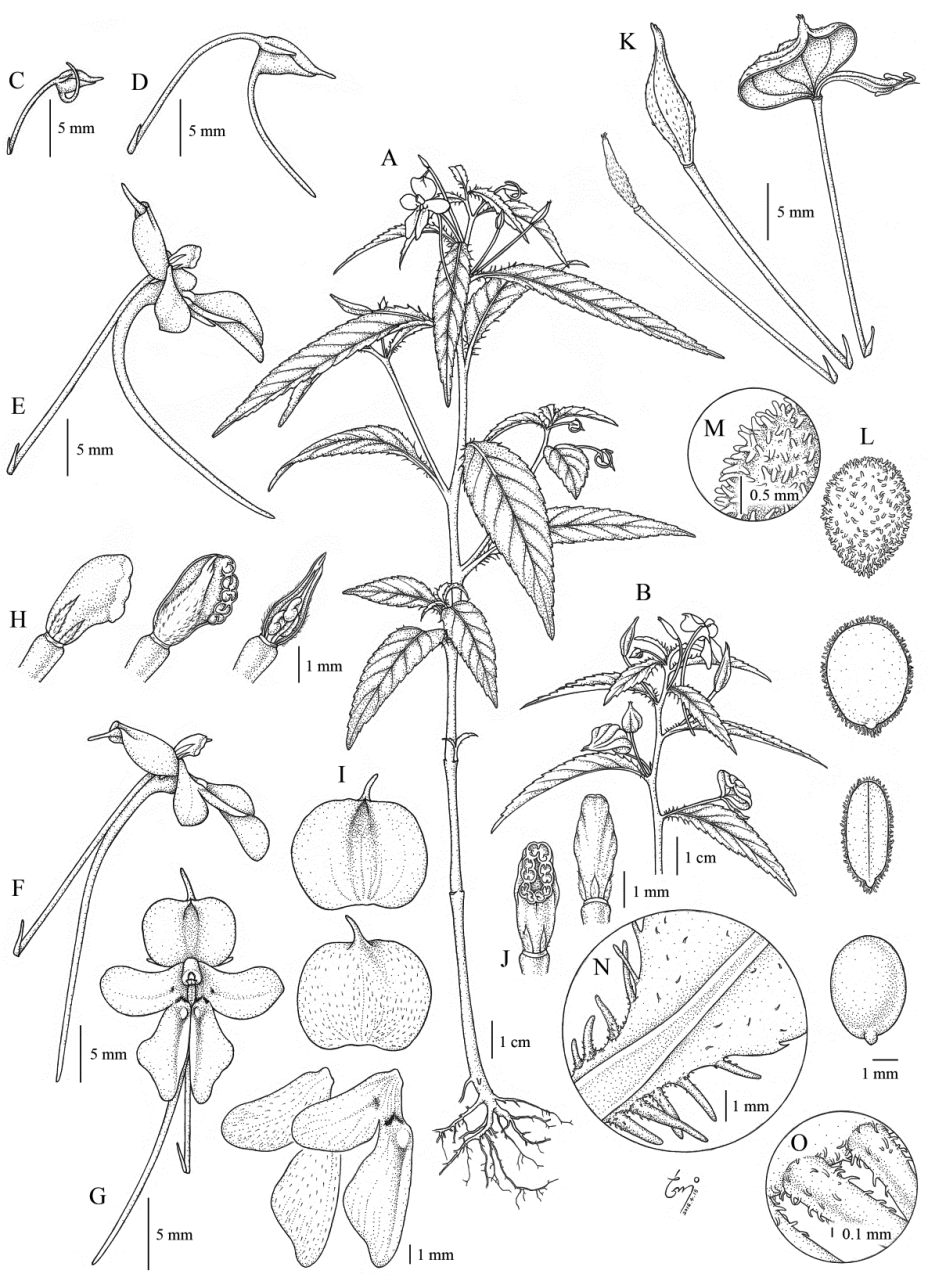
*Impatiens bokorensis* **A** Flowering individual **B** Fruiting individual **C–D** Developing flower bud **E** Mature flower (before pollination) **F–G** Mature flower (after pollination) **H** Developing gynoecium **I** Petals **J** Androecium **K** Developing fruit **L–M** Seed **N–O** strigose-ciliate at leaf base: Cho et al. CB-3112, 3432. Illustration by Hye-Woo Shin.

**Figure 2.**
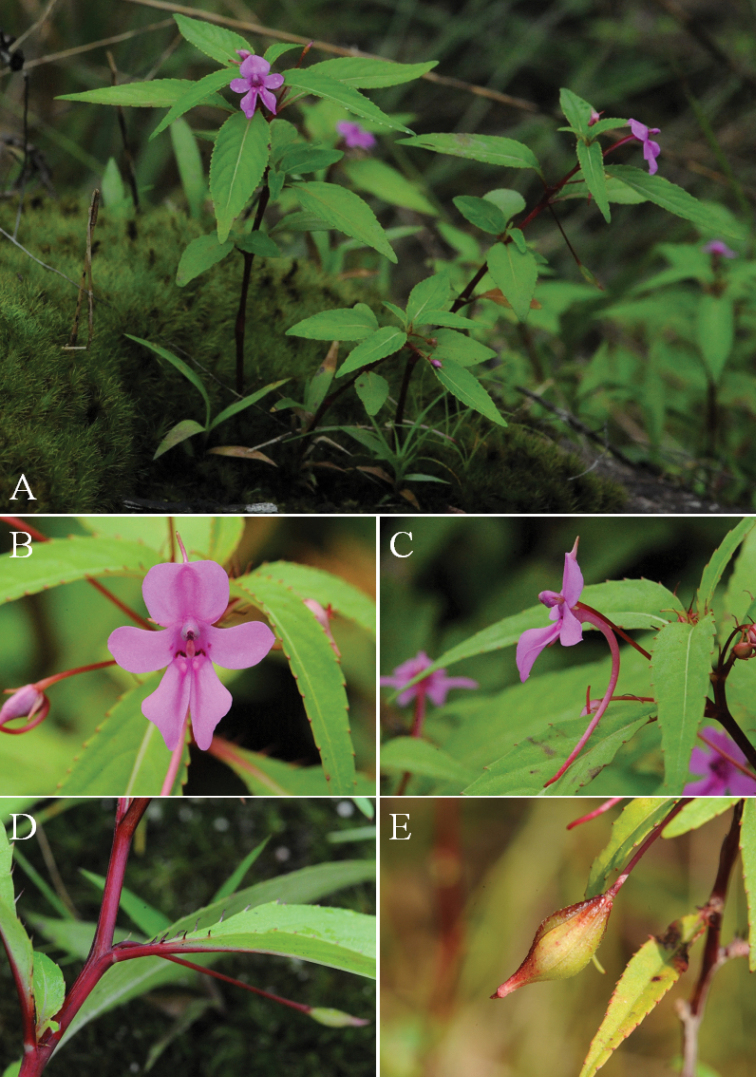
**A–E** *Impatiens bokorensis* **A** Habit **B–C** Flower **D** strigose-ciliate at leaf base **E** Capsule: Photos by Seong-Hyun Cho.

During the recent floristic survey, one species of *Impatiens* was collected at Bokor National Park in Southwestern Cambodia that does not appear to be similar to previously reported species (Figures 1 & 2). It is most similar to *Impatiens patula* Craib from Thailand (Craib 1926, Shimizu 1970), but a comparison with the type specimens and descriptions revealed that it differs from *Impatiens patula* and is therefore described here as a new species.

## Taxonomy

### Impatiens bokorensis

Taxon classificationPlantaeEricalesBalsaminaceae

S.H.Cho & B.Y.Kim sp. nov.

urn:lsid:ipni.org:names:77160179-1

Figures 1 , 2

#### Type.

CAMBODIA. Kampot Province, Phnum Bokor National Park, sandstone tables in evergreen forest margin, 10°38'20.8"N, 104°00'16.0"E, a.s.l. 1,050 m, 24 August 2015, with flowers, Cho S.H, Kim B.Y., Park H.S., Chhang Phourin CB-3112 (holotype HHU!, isotypes KB!, KRIB!, RUPP!).

#### Diagnosis.

*Impatiens bokorensis* is most similar to the Thailand endemic species *Impatiens patula* Craib in habit but is readily distinguished from the latter by the orbicular-obovate dorsal petal, shorter pedicels and larger seeds (Table 1).

**Table 1.** Comparison of key features of *Impatiens bokorensis* and *Impatiens patula*.

Taxonomic traits	*Impatiens bokorensis*	*Impatiens patula*
Leaf		
upper surface	pubescent	pubescent with scurfy hairs
lower surface	glabrous to sparsely pubescent	pubescent on nerves only or tomentose all over
strigose-ciliate at base	1–2.7 mm	1–1.4 mm
Pedicel	1.4–2.0 cm	2.3–3(–5) cm
Dorsal petal	orbicular-obovate, ca. 6 × 8 mm, horned at apex, horn 2.2–2.3 mm long	cordate, 7 mm long, horned at apex, horn 3 mm long
Seeds	3.8–4.6 × 2.6–3.2 mm	3.5 × 2.5 mm

#### Description.

Herbs, annual, terrestrial, hermaphroditic. Stems erect, 15–40 cm tall, tinged purplish red, branched, glabrous or sparsely puberulous on the upper part. Leaves simple, alternate; petioles subsessile to 1.4 mm; leaf blade lanceolate to ovate-lanceolate, apex acuminate, base narrowly cuneate to attenuate, 2.5–7.0 × 0.6–2.0 cm, upper surface pubescent, lower surface glabrous to sparsely pubescent, secondary veins pinnate, 6 to 8 on each side of mid-vein, margin serrate, teeth mucronate and purple tinged, strigose-ciliate at base; strigose-ciliate, 4–14, 1–2.7 mm long, purple to purplish black, minutely puberulous at base. Flowers axillary, solitary, rarely 2 fascicled, zygomorphic, minutely puberulous; pedicels slender, erect, purplish red, 1.4–2.0 cm long, glabrous, bracteate at base; bracts linear, up to 4 mm; lateral sepals 2, linear-lanceolate, 2.5–3 mm long, glabrous; lower sepal funnel-form, pink, ca. 5 mm long, ca. 3 mm deep; spur 17–23 mm long, slightly curved; dorsal petal, orbicular-obovate, ca. 6 × 8 mm, horned at apex, horn 2.2–2.3 mm long; lateral united petals separate, bilobed, ca. 11 mm long; upper petals oblong, 6.0–6.5 × ca. 3.0 mm, minutely apiculate; lower petals, 9.0–9.3 × 3.5–3.8 mm; androecium ca. 2.8 × 1.6 mm; stamens 5, connate, surrounding gynoecium; filaments ca. 0.7 mm; ovary fusiform, pubescent, ca. 2.5 × 1.0 mm; style glabrous, ca. 0.2 mm long; stigma 5, ca. 0.25 mm long. Fruit a capsule, fusiform, ca. 15 × 6 mm, pubescent with scurfy hairs, 3[4]-seeded. Seeds obovoid, slightly compressed, 3.8–4.6 × 2.6–3.2 mm, pubescent with spirally sculptured hairs.

#### Specimen examined.

CAMBODIA. 16 November 2015, with fruits, Cho et al. CB-3432 (HHU!, KRIB!); 2 September 2016, with flowers, Kim et al. CB-3537 (HHU!)

#### Phenology.

Flowering specimens were collected in August and fruiting specimens in November.

#### Distribution and habitat.

*Impatiens bokorensis* grows on sandstone tables in evergreen forest margins at 1,050 m a.s.l.. Endemic to southwestern Cambodia, *Impatiens bokorensis* is at present known only in the type locality.

#### GenBank Accession No.

Cho et al. CB-3432: KX171761 (ITS).

#### Conservation status.

*Impatiens bokorensis* was collected in Phnum Bokor National Park in southwestern Cambodia. Until now, only one population, consisting of ca. 200 individuals, has been discovered in the park area; therefore, it is preliminarily classified as data deficient (DD) according to the IUCN Red List criteria (IUCN 2001).

## Supplementary Material

XML Treatment for Impatiens bokorensis
